# Complex Cells in the Brain's Vision Center Tune in to Natural Scenes

**DOI:** 10.1371/journal.pbio.0030360

**Published:** 2005-09-27

An Amur tiger roaming the snow-covered forests of the Russian Far East sees life differently than an arboreal primate raised in the thick canopy of a tropical jungle. Adaptations in the structure of the eye and the visual centers of the brain facilitate these different worldviews, fine-tuning each animal's vision to the light levels and visual properties of its environment.

The notion that brain circuits are adapted to represent natural stimuli is known as the efficient coding hypothesis. In this framework, the visual system responds most effectively to the features found in the natural environment, like the specific arrangement of trees, another animal's face, or the contrast of land abutting a river. Natural images possess different statistical features than random noise (such as static on a TV monitor), so responses to these two classes of stimuli could be distinct. According to the prevailing hypothesis, the retina and the lateral geniculate nucleus, the brain area that relays neural signals to the cortex, are tuned to the power spectrum of light signals found in natural scenes. In other words, these neurons are sensitive to the dominant energy at particular spatial or temporal frequencies in natural scenes and less sensitive to random noise, which has an overall flat power spectrum.

In a new study, Gidon Felsen, Yang Dan, and colleagues investigate how neurons in the cat primary visual cortex (V1) respond to images with natural statistics, and discover something truly novel about a V1 neuron's sensitivity to features in natural scenes: a specific class of V1 neurons, called complex cells, are preferentially tuned to the phase regularities of light signals in natural scenes, not to the power spectrum. (Phase relates to how the signals in different spatial frequencies align, which gives rise to edges in the image.)[Fig pbio-0030360-g001]

**Figure pbio-0030360-g001:**
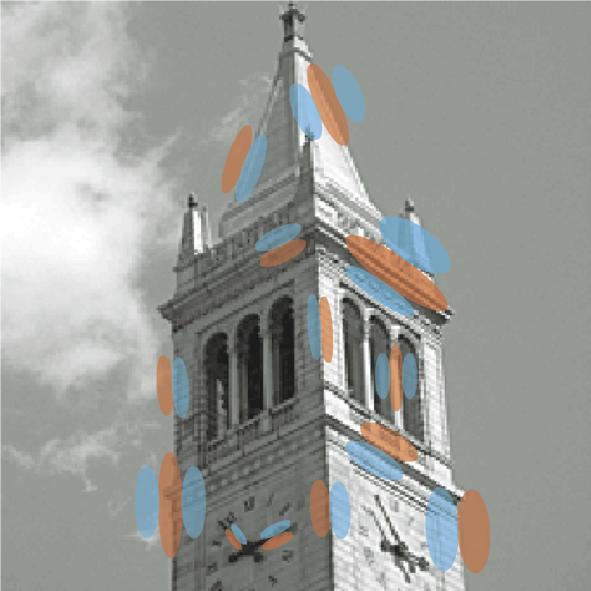
By recording cortical responses to several classes of natural and synthetic images, researchers showed that complex cells are tuned to the phase structure of natural images to represent image features, such as the edges highlighted on the clock tower, efficiently

The V1 contains two types of neurons, known as simple and complex cells, that were originally distinguished based on how they respond to light, determined by shining a flashlight on a wall and mapping the space that triggered neuronal activation. This activation space defines the cell's receptive field. The receptive field properties of simple and complex cells vary significantly: simple cells behave linearly (two spots of light in the receptive field doubles the response), and complex cells behave nonlinearly.

To characterize the feature sensitivity of these neurons, the authors measured their response to different classes of visual stimuli, including natural, random, and synthesized images; the synthesized images helped them distinguish between the power spectrum and phase effects. Feature sensitivity depends on a neuron's preferred features, which the authors estimated from a neuron's response to a set of natural images, including a man's face, a building, a lion, and a hand. The authors created a set of random images with global and feature contrasts that matched the natural images, based on the preferred features, and recorded neuronal response to both the natural and random image sets. Overall, the majority of complex cells showed higher feature sensitivity for the natural stimuli, indicated by the contrast-response function, which plots neuronal response against the contrast of the feature. (A steep contrast-response function shows high neuronal sensitivity to a preferred feature, while a flat function indicates insensitivity.) Simple cells showed no difference in their response to natural and random stimuli.

Since the contrasts of natural and random image sets were matched, the complex cells' sensitivity to natural images could not be explained by differences in overall contrast. More likely, the authors reasoned, the cells were responding to the power or phase spectra of the light. Felsen et al. manipulated each property in synthesized image sets to distinguish their effects on neuronal response. In one image set, each image had a natural power and random phase spectrum, while a second image set had the reverse. Comparing the feature sensitivity of complex cells to each of these image sets with a random image set, the contrast-response function, and thus feature sensitivity, was highest for the synthesized natural phase image set.

By experimentally linking visual statistics with neuronal responses, this study not only reveals a novel coding response property of complex cells but also provides evidence for the theory of efficient coding. The finding that complex cells selectively respond to properties of natural stimuli that simple cells don't shows how brain circuits divide tasks to make the most of available resources. Researchers can now investigate how the structure of complex cells engenders their heightened sensitivity. —*Liza Gross*

